# COVID-19: Pandemic Risk, Resilience and Possibilities for Aging Research

**DOI:** 10.1017/S0714980820000215

**Published:** 2020-05-19

**Authors:** Andrew Wister, Mark Speechley

**Affiliations:** 1 Department of Gerontology, Simon Fraser University, Vancouver, British Columbia; 2 Department of Epidemiology and Biostatistics, Schulich Interfaculty Program in Public Health, Western University, London, Ontario.

**Keywords:** vieillissement, COVID-19, géro-pandémie, personnes âgées, vulnérabilité, résilience, aging, COVID-19, gero-pandemic, older adults, vulnerability, resilience

## Abstract

The COVID-19 global crisis is reshaping Canadian society in unexpected and profound ways. The significantly higher morbidity and mortality risks by age suggest that this is largely a “gero-pandemic,” which has thrust the field of aging onto center stage. This editorial emphasizes that vulnerable older adults are also those most affected by COVID-19 in terms of infection risk, negative health effects, and the potential deleterious outcomes on a range of social, psychological, and economic contexts – from ageism to social isolation. We also contend that the pathogenic analysis of this pandemic needs to be balanced with a salutogenic approach that examines the positive adaptation of people, systems and society, termed *COVID-19 resilience.* This begs the question: how and why do some older adults and communities adapt and thrive better than others? This examination will lead to the identification and response to research and data gaps, challenges, and innovative opportunities as we plan for a future in which COVID-19 has become another endemic infection in the growing list of emerging and re-emerging pathogens.

## RÉSUMÉ

La crise mondiale de la COVID-19 remodèle la société canadienne de manière inattendue et profonde. Les risques significativement plus élevés de morbidité et mortalité en fonction de l’âge suggèrent qu’il s’agit en grande partie d’une «géro-pandémie», qui a propulsé le domaine du vieillissement à l’avant-scène. Cet éditorial souligne que les personnes âgées vulnérables sont également les plus touchées par la COVID-19 en termes de le risque d’infection, d’effets négatifs sur la santé et de conséquences délétères potentielles sur une gamme de facteurs sociaux, psychologiques, économiques - de l’âgisme à l’isolement social. Nous soutenons également que l’analyse pathogène de cette pandémie doit être équilibrée avec une approche salutogène qui examine l’adaptation positive des personnes, des systèmes et de la société, appelée Résilience COVID-19. Ceci soulève la question: comment et pourquoi certaines personnes âgées et communautés s’adaptent et réussissent mieux que d’autres? Cet examen mènera à l’identification et à la réponse aux lacunes dans la recherche et les données, aux défis et aux opportunités innovantes alors que nous planifions un avenir dans lequel la COVID-19 est devenue une autre infection endémique dans la liste croissante des pathogènes émergents et ré-émergents.

The COVID-19 global crisis has occurred swiftly with far-reaching consequences in terms of public health, economies, communities and families. The differential mortality risks suggest that this is largely a “gero-pandemic,” which has brought the field of aging into center-stage, in both pathogenic and salutogenic contexts.

Canadian older adults are highly diverse, and generally, healthy, engaged and active. Yet, there are high risk groups of older adults who are the most affected by COVID-19 in terms of both infection risk and negative health outcomes, especially mortality. Older adults living in congregate living environments, including long-term care, supportive housing or assisted living, are exposed to increased infection risk from living in institutions designed around short physical distancesamong residents. Infection risk is exacerbated when staff are required to re-use personal protective equipment because of shortages, and still further when low level staff are so poorly paid they must have jobs at multiple sites. The outcomes of institutionalized older adults are worse in large measure because of the high prevalence of multimorbidity. However, many seniors living in the community also face elevated morbidity and mortality risks. Even those not experiencing COVID-19 face challenges meeting their basic needs such as obtaining food, medications, and health care. Some of the most vulnerable subgroups of older adults are those with physical or mental health challenges and mobility restrictions; those living in poverty; alone or with few informal supports; and those providing care for another without the needed resources.

As of the second week of June, 2020, COVID-19 cases are approaching 100,000 in Canada and surpassing 7 M worldwide; and these are underestimates because of the current testing limitations. The number of deaths exceeds in 7,800 Canada and 400,000 globally (government of Canada). Thus far, counts of new cases and deaths are usually available disaggregated by age, sex, health region, province and country; outbreak hot-spots; and hospital rates (including intensive care units). A significant amount of attention has been on age. Approximately 36 per cent of positive cases are among persons 60 years and older in Canada; however,approximately 90 per cent of deaths are among this group. The median age of COVID-19 diagnosis is approximately 42; the median age of death is a notable 86, with more than half of all deaths occurring in long-term care facilities. The bulk of Canadian cases comprise the baby boomers - persons born between 1946 and 1965 (aged about 55-74 years of age today) - plus the smaller generation born during the Great Depression who are now 75 years and over. Although data are available on pre-exiting conditions, such as cardiovascular disease, cancer, diabetes, and chronic obstructive pulmonary disease, we know little about the longer-term health consequences for those who recover from the disease, and the broader older population.

Furthermore, more detailed data for other forms of inequality and social deprivation, such as race, ethnicity, income, education, work status, sexual orientation, and living arrangement are not currently available. This has placed attention on age and to a lesser degree sex (55 per cent of cases are female, probably because they are more likely to be tested, and/or have genetic, immune system or hormonal protection). Although COVID-19 knows no borders, physical or social, it has clearly become an aging-related disease. On the one hand, gerontologists have already become important contributors to COVID-19 knowledge, practice and research. On the other hand, there is a backlash of younger and working populations, fed by media and political hype, who believe that they are less susceptible, and if they do become infected, the symptoms will be less serious than for older populations. Some of these views have been articulated as part of the "ok boomer" movement, which has pitted younger and older generations against each other. Other individuals and groups have expressed the view that the COVID-19 pandemic is largely a “seniors problem” and as such should not shut down the economy and society to the level that has occurred. Some politicians have even gone so far as to suggest that older people ought to consider sacrificing themselves for the health of others, including that of the economy.

Older persons comprise the bulk of COVID-19 cases, as with many other infectious and chronic diseases. However, focusing on older patients conveniently ignores the number of younger adults including scores of frontline healthcare workers who have also fallen ill. Unfortunately, as soon as the COVID-19 pandemic is presented in stark generational terms it has the potential to significantly accentuate ageism in society. In 1968, the public housing authority in Chevy Chase, Maryland applied to convert a building in a white, middle-class suburb into housing for older citizens. The public hearings degenerated into a riot as residents of the area fought to keep “all those old people” out of their community. As a result of this incident, Butler (1969) coined the term “ageism.” Butler defined ageism as a process of systematic stereotyping of and discrimination against people because they are old. He considered ageism to be similar to racism and sexism in that inherent biological factors are used to define personality or character traits. Ageism can be expressed, fostered, and perpetuated by the media, by public policies, in the workplace, and in casual daily interactions with older people. Where ageism exists, older people are devalued, and their human rights are compromised. In short, there is both *individual* ageism, the acceptance of negative feelings and beliefs that influence our thinking and behaviour, and *institutionalized* (or structural) ageism, as expressed in legislation and mass media, all of which can lead to social and economic inequalities across society (Bytheway 2005). The many ways in which this pandemic will influence ageism and discrimination require attention by those studying human aging.

The COVID-19 crisis has resulted in our communities coming together to leverage medical, social and technological resources. Families, friends, and many community groups, often relying on volunteers (including gerontology students), are providing supports to older adults and their families to meet basic needs in a safe manner. Although there are technological solutions available to assist people to remain connected to others, and obtain necessay products and services, there is a digital divide, such that many older adults do not have access to or the ability to use smart phones, tablets, or computers. Older “low tech” solutions like the telephone may be more effective in reaching the most vulnerable older adults, such as those who live alone. The government, health authorities, and non-governmental organizations are also taking important steps to serve the needs of seniors during the COVID-19 crisis. For example, policy changes to restrict health workers from working at more than one long term care facility, and ramped up testing of these workers, are reducing the risk and spread of the disease in these particularly at-risk facilities.

We also need to learn from this crisis for the future, especially examining the positive adaptation of people and society – what we might call *COVID-19 resilience.* This begs the question: how and why do some older adults adapt and thrive better than others? This question addresses those who have experienced the disease firsthand and those who experience the social (isolation), psychological (chronic stress) and economic (loss of income) outcomes of COVID-19. The enormous variation in risk and response to the disease suggests that aging-related intra-variability may be as important as comparing ages or age groups. COVID-19 resilience occurs at the individual, family, community, and societal/system level. At the individual level, resilience models can be useful in understanding the role of cycles of disruption and reintegration in rebalancing health and well-being, and the key resources that need to be available and harnessed to help individuals bounce back from adversity (Wister et al., 2016). Resilience and aging research has established support for the importance of emotional/positive affect (Ong et al., 2006), social/family support (Rybarczyk, 2012), health behaviours (Wister et al., 2019), community connectedness (Wiles et al., 2012), and system level components of disaster resilience (Linkov and Kott, 2019). Applications of this research to the COVID-19 pandemic has potential to balance the deleterious consequences with positive adaptation and growth.

We need to continue to develop innovative ways to provide the necessary supports to older adults most at risk, and to learn from those most resilient. Most older adults are healthy and were previously socially connected; however, in the COVID era, like everyone else, they face the adverse effects of stress and isolation. Social problems that many older people currently face, such as elder abuse, social isolation, loneliness, anxiety, depression, food insecurity, and spousal caregiving burden, may be exacerbated by COVID-19. Understanding the nexus of risk, vulnerability, and resilience has potential to identify key questions and shape a path forward, including but not limited to the following. How and why are some individuals, families, and communities able to adapt to a COVID-19 era? What can we learn – both positive and negative - from societies with large proportions of older persons, such as Italy and Japan? What are the most important resilience resources (e.g., family/community supports)? What policies have worked (or not worked) to reduce risk and enhance resilience (e.g., long term care policies for health workers)? How has social isolation and loneliness affected older people and their families? What system-level preparedness, responses and adaptations have promoted the most positive responses to the pandemic? How has ageism and discrimination been affected by COVID-19? And how can we be better prepared for future disasters? Surveillance, innovative research, and training are fundamental. New data, such as those collected in a COVID-19 sub study, part of the ongoing Canadian Longitudinal Study of Aging (www.clsa-elcv.ca), (following more than 51,000 Canadians for 20 years and collecting interdisciplinary data from cell to society) will support this work. Under the leadership of principal investigators, P. Raina, C. Wolfson and S. Kirkland, and the collaboration of 11 universities and over 150 researchers across Canada, the CLSA is positioned to fill knowledge gaps pertaining to the epidemiology, social, psychological, and economic aspects of this pandemic.

This crisis has provided new opportunities by mobilizing the community of aging-related basic and applied researchers, as well as the spectrum of program and policy makers, to generate innovation in knowledge and its translation.

## References

[r1] Butler, R. (1969). Ageism: Another form of bigotry. The Gerontologist, 9(3), 243–246. 5366225 10.1093/geront/9.4_part_1.243

[r2] Bytheway, B. (2005). Ageism and age categorization. Journal of Social Issues, 61(2), 361–374.

[r3] Government of Canada. (2020). *Coronavirus Disease (COVID-19).* http://www.canada.ca/en/public-health/services/diseases/coronavirus-disease-covid-19.html. Retrieved May 14, 2020.

[r4] Linkov, I., & Kott, A. (2019). Fundamental concepts of cyber resilience: Introduction and overview In Cyber Resilience of Systems and Networks (pp. 1–25). New York: Springer.

[r5] Ong, A. D., Bergeman, C. S., Bisconti, T. L., & Wallace, K. A. (2006). Psychological resilience, positive emotions, and successful adaptation to stress in later life. Journal of Personality and Social Psychology, 91(4), 730–749. 17014296 10.1037/0022-3514.91.4.730

[r6] Rybarczyk B. , Emery, E., Guequierre, L., Shamaskin, A. & Behel, J. (2012) Resilience and family caregiving In B. Hayslip and G. Smith, Eds, Annual Review of Gerontology and Geriatrics, Volume 32, 2012, Special Issue: Emerging Perspectives on Resilience in Adulthood and Later Life, 173-188. New York: Springer.

[r7] Wiles, J., Wild, K., Kerse, N., & Allen, R. (2012). Resilience from the point of view of older people: There’s still life beyond the funny knee. Social Science and Medicine, 74, 416–424. 22204841 10.1016/j.socscimed.2011.11.005

[r8] Wister, A., Coatta, K., Schuurman, N., Lear, S., Rosin, M., & MacKey, D. (2016). A Lifecourse model of multimorbidity resilience: Theoretical and research developments. *International Journal of Aging & Human Development.*, 82(4), 290–313. doi:10.1177/0091415016641686. 27076489

[r9] Wister, A., Cosco, T., Mitchell, B., & Fyffe, I. (2019). Health behaviors and multimorbidity resilience among older adults using the Canadian Longitudinal Study on Aging. International Psychogeriatrics. First View 1-15. doi:10.1017/S104161021900048. 31088579

